# Minimal change nephrotic syndrome in patients infected with human immunodeficiency virus: a retrospective study of 8 cases

**DOI:** 10.1186/s12882-018-1132-x

**Published:** 2018-11-20

**Authors:** Romain ARRESTIER, Anne-Pascale SATIE, Shao-yu ZHANG, Emmanuelle PLAISIER, Corinne ISNARD-BAGNIS, Philippe GATAULT, Quentin RAIMBOURG, David BUOB, Flavia VOCILA, Anne-Elisabeth HENG, Helene FRANCOIS, Anissa MOKTEFI, Guillaume CANAUD, Marie MATIGNON, Nathalie DEJUCQ-RAINSFORD, Isabelle BROCHERIOU, Dil SAHALI, Vincent AUDARD

**Affiliations:** 1 AP-HP (Assistance Publique-Hôpitaux de Paris), Service de Néphrologie et de Transplantation, Centre de Référence Maladie Rare Syndrome Néphrotique Idiopathique, Hôpital Henri-Mondor/Albert-Chenevier, F-94000 Créteil, France; 2 Université Paris-Est Créteil (UPEC), UMR-S955, F-94000 Créteil, France; 3 Institut National de la Santé Et de la Recherche Médicale (INSERM), U955, équipe 21, F-94000 Créteil, France; 4 Irset (Institut de recherche en santé, environnement et travail) – UMR_S 1085, Univ Rennes, Inserm, EHESP (Ecole des Hautes Etudes en Santé Publique), F-35000 Rennes, France; 5 Sorbonne Université, AP-HP, Service de Néphrologie, Centre de Référence Maladie Rare Syndrome Néphrotique Idiopathique, Hôpital Tenon, F-75020 Paris, France; 6 AP-HP, Service de Néphrologie, Hôpital de La Pitié Salpêtrière, F-75013 Paris, France; 7 Service de Néphrologie et Transplantation, Hôpital Bretonneau, F-37000 Tours, France; 8 AP-HP, Service de Néphrologie, Hôpital Bichat, F-75018 Paris, France; 9 AP-HP, Service d’Anatomie Pathologique, Hôpital Tenon, F-75020 Paris, France; 10 Service de Néphrologie Centre Hospitalier de Cannes, F-06400 Cannes, France; 11 Service de Néphrologie, Dialyse, Transplantation, CHU (Centre Hospitalier Universitaire) Clermont-Ferrand, UMR 1019, INRA (Institut National de la Recherche Agronomique), Université Clermont Auvergne, Clermont-Ferrand, France; 12 AP-HP, Service Médecine Interne et Immunologie clinique, Hôpital Bicêtre, F-94275 Le Kremlin-Bicêtre, France; 13 AP-HP, Service d’Anatomie Pathologique, Hôpital Henri-Mondor/Albert-Chenevier, F-94000 Créteil, France; 14 INSERM U1151, Institut Necker Enfants Malades, Hôpital Necker-Enfants Malades, Paris, France; 15 Université Paris Descartes, Sorbonne Paris Cité, Hôpital Necker-Enfants Malades, Paris, France; 16 AP-HP, Service de Néphrologie Transplantation Adultes, Hôpital Necker-Enfants Malades, Paris, France; 17 AP-HP, Service d’Anatomie Pathologique, Hôpital de La Pitié Salpêtrière, F 75013 Paris, France

**Keywords:** Minimal change nephrotic syndrome, HIV infection, AIDS, Podocytes, Albuminuria, C-mip, Rituximab

## Abstract

**Background:**

Human immunodeficiency virus (HIV) is associated with diverse glomerular diseases. Characteristics of minimal change nephrotic syndrome (MCNS) in this setting have been little studied, and the specific features of this uncommon association remain to be determined.

**Methods:**

We conduct a retrospective study. Clinical, biological and pathological characteristics of patients with MCNS and HIV infection were assessed. We evaluated HIV infection by in situ hybridization and CMIP expression by immunochemistry on kidney biopsies and compared it to HIV-associated nephropathy (HIVAN) and idiopathic MCNS.

**Results:**

Eight patients were identifies. In all but one of these cases, MCNS occurred after HIV diagnosis (mean of 9.5 years). Acute kidney injury was detected in three cases. Mean CD4^+^ lymphocyte count was 733/mm^3^ and three patients had a detectable HIV viral load. In situ hybridization for HIV-1 RNA detection yielded a positive signal in a few tubular cells in the renal parenchyma in two of four patients with HIV infection associated with MCNS. Podocytes of these patients presented strong positive immunostaining for CMIP (4/4). Three patients suffered steroid-dependent nephrotic syndrome, and another two patients had at least one relapse. Rituximab treatment was initiated in four cases. After a median follow-up of 20 months, all patients were in remission (complete in 5 cases).

**Conclusions:**

In patients with MCNS occurring in a context of HIV infection, podocyte injury seems to be associated with CMIP induction rather than renal HIV infection but further studies are needed to determine the molecular link between these two conditions.

## Background

Minimal change nephrotic syndrome (MCNS) is a glomerular disease characterized by massive selective proteinuria without glomerular lesions on light microscopy and with no immunoglobulin deposits on immunofluorescence study. MCNS has a higher incidence in children than in adults, but is, nevertheless, a frequent cause of nephrotic syndrome (NS) in adults, accounting for 10 to 25% of cases [1]. The pathogenesis of MCNS remains poorly understood, but there is compelling evidence, to suggest that it involves both immune system impairment and podocyte dysfunction [2]. In most cases, MCNS is considered to be an idiopathic glomerular disorder potentially triggered by immunological stimuli, such as viral infection, immunization or allergens [2]. MCNS may occur in association with chronic lymphoid neoplasms, such as Hodgkin disease [3], non-Hodgkin lymphoma [4] and thymoma [5], consistent with the hypothesis that it results from an immune system disorder. In the course of studies on molecular mechanisms underlying the pathophysiological processes involved in MCNS occurrence, we originally identified CMIP (c-maf inducing protein) as induced in T lymphocytes of patients with MCNS relapse but further studies have shown that CMIP is overproduced in podocytes of these patients, whereas it is downregulated in both tissues in MCNS remission [6, 7]. In addition, transgenic mice expressing selectively CMIP in podocytes develop heavy proteinuria without inflammatory lesions or immune complex deposits [6] and there is growing evidence that increase CMIP abundance could dramatically affect the function and survival of podocytes [2]. In rare cases, MCNS has also been reported in association with chronic viral infection [8, 9]. Human immunodeficiency virus (HIV) infection is a well-known cause of renal disease [10]. HIV-associated nephropathy (HIVAN) is the most common cause of glomerular injury in these patients [11]. A large number of studies have clearly demonstrated that HIVAN pathogenesis is triggered by the direct infection of glomerular epithelial cells and tubular epithelial cells by HIV [12]. In some studies, MCNS has been reported to account for 2–4% of all nephropathies associated with HIV [13, 14]. An extensive review of seven renal pathology studies including a total of 949 patients showed MCNS to be the main renal biopsy finding in 10 patients (1.05% of cases), suggesting that MCNS is a rare finding in HIV-infected patients [11]. It remains unclear whether there is a real pathophysiological link between MCNS and HIV infection, or just a fortuitous association.

In this study, we retrospectively identified patients with biopsy-proven MCNS and diagnosed with HIV infection, and we analyzed their clinical, histological, laboratory, and treatment data, to assess the significance of this rare association. We also performed in situ hybridization to determine whether HIV-RNA could be detected on renal biopsies from these patients and to assess possible CMIP expression, which has recently been shown to be associated with podocyte dysfunction [6].

## Methods

### Patients

We conducted a retrospective study, by sending a questionnaire to all French nephrology departments, to investigate the clinical, biological pathological and therapeutic data of adult patients with biopsy-proven MCNS in a context of HIV infection. At each hospital, patients were identified by searching renal pathology and clinical diagnosis databases. Eight patients were identified, for whom data were collected between 2000 and 2016, at the nephrology departments of eight French hospitals (Henri Mondor, Kremlin-Bicêtre, Pitié-Salpêtrière, Bichat, Tenon, Cannes, Clermont Ferrand and Tours). The study was performed in accordance with the ethical standards of the Helsinki Declaration, and has been approved by our local institutional review board.

Data were assessed for each patient at the time of MCNS diagnosis. The demographic and comorbid data collected included age, sex, ethnicity, hypertension, and coinfections with hepatitis B or hepatitis C viruses. Time from the diagnosis of HIV infection to the occurrence of MCNS, previous opportunistic infections, CD4^+^ T-cell count and HIV viral load were also recorded.

Highly active antiretroviral therapy (HAART) at the time of MCNS diagnosis was reported. All patients underwent renal biopsy for the exploration of a nephrotic syndrome, defined as a urinary protein excretion rate of more than 3 g/day and a serum albumin concentration below 30 g/L. MCNS diagnosis was confirmed by a pathological examination showing the presence of minimal change glomerular lesions on light microscopy, with no immunoglobulin and/or complement deposits in immunofluorescence study. Patients diagnosed with HIVAN on the basis of renal biopsy, or with focal and segmental glomerulosclerosis (FSGS) lesions without the typical pathological features of HIVAN were excluded. Acute kidney injury (AKI) was defined according to KDIGO (kidney disease improving global outcome) criteria [15]. Chronic kidney disease was defined as a permanent (lasting at least three months) decrease in estimated glomerular filtration rate (eGFR) to less than 60 mL/min/1.73 m^2^, according to the Chronic Kidney Disease Epidemiology Collaboration (CKD-EPI) formula [16]. Changes in HAART after MCNS diagnosis were systematically monitored. Complete remission (CR) of MCNS was defined as the return of urinary protein concentration to the normal range (< 0.3 g/24 h), together with an albumin level > 30 g/L. Relapse was defined as the recurrence of proteinuria in the nephrotic range for more than one week. Patients were considered to be in partial remission (PR) if proteinuria was between 0.3 and 3 g/day and albumin concentrations above 30 g/L. Steroid-dependent and steroid-resistant nephrotic syndromes were as previously defined by Hogan et al. [1].

### In situ hybridization

The potential localization of HIV infection in renal tissues affected by MCNS, as previously described [17], was investigated by in situ hybridization (ISH) for HIV-1 RNA on alcohol-formalin-acetic acid solution–fixed paraffin-embedded tissues from kidney biopsies. Labeling specificity was systematically checked by hybridizing sense probes with adjacent sections in parallel and antisense probes with uninfected renal tissues. For ISH, two patients with biopsy-proven HIVAN were used as positive controls (both have low CD4^+^ T-lymphocytes count < 200/mm^3^ and detectable viral load at the time of renal biopsy) and two patients with idiopathic MCNS as negative controls (initial episode in both cases).

### Immunohistochemical analysis of CMIP expression

For immunohistochemistry-based analyses of CMIP expression, kidney samples were fixed for 16 h in Dubosq Brazil, then dehydrated and embedded in paraffin. Antigen retrieval was performed by immersing the slides in 0.01 mol/L citrate buffer and heating them in a 500 W microwave oven for 15 min. Endogenous peroxidase activity was blocked by incubation with 0.3% H_2_O_2_ in methanol for 30 min. Slides were incubated with blocking reagents containing avidin-biotin solution for 30 min and with normal blocking serum for 20 min, and were then incubated overnight with polyclonal anti-CMIP antibody at a final concentration of 2.5 μg/mL.

## Results

### Clinical and biological data for patients with MCNS occurring in a context of HIV infection

We retrospectively identified eight patients (three men and five women) with a diagnosis of MCNS and HIV infection. Their clinical and biological data at the time of renal biopsy are summarized in Table 1. Mean age at MCNS diagnosis was 43.3 years (range: 20 to 66 years) and five patients were of African ancestry. MCNS occurred after the diagnosis of HIV infection in seven cases, with an estimated mean interval of 9.5 years between these two events. In the remaining patient, the diagnosis of HIV infection coincided with MCNS (patient 8). All patients had typical features of MCNS at diagnosis, with nephrotic syndrome in all cases (mean proteinuria of 7.96 g/day (range: 3.33 to 14.8 g/day) and a mean albumin concentration of 17.5 g/L (7.7 to 29.5 g/L). None of them had MCNS resulting from a secondary process (lymphoid disorders or malignant disease) or potentially related to treatment known to be associated with MCNS occurrence (Lithium, Interferon, non-steroidal anti-inflammatory drugs). Three patients (37.5%) displayed AKI according to KDIGO criteria (stage 1 in two cases and stage 2 in one case). Four patients displayed microscopic hematuria during the first episode of MCNS (patients 2, 4, 5 and 7) and 1 patient (patients 3) presented microscopic hematuria during at least one relapse. At the time of renal biopsy, all but one (patient 8) of the patients had already received HAART, resulting in the successful control of CD4^+^ T-lymphocyte counts (mean CD4^+^ T-lymphocyte count of 733/mm^3^, range: 413/mm^3^ to 1028/mm^3^), but HIV was nevertheless detectable in three patients (patients 1, 7 and 8). As expected, HIV viral load was highest in patient 8, whose HIV infection was newly diagnosed (47,263 copies/mL). In this patient, HAART was not introduced immediately after HIV diagnosis because CD4^+^ T-lymphocytes count was normal (480/mm^3^).

**Table 1:** Baseline characteristics of patients at the time of MCNS diagnosis

Patient	1	2	3	4	5	6	7	8
Sex	F	M	F	F	F	M	F	M
Age (years)	33	66	42	50	47	56	33	20
Ethnicity	African	Caucasian	Caucasian	African	African	Caucasian	African	African
HT	No	No	No	No	No	Yes	No	No
Time between HIV diagnosis and MCNS (yr)	7	7	22	9	4	23	4	0
CD4^+^ T-cell count at MCNS diagnosis (/mm^3^)	1028	738	1027	918	413	811	450	480
HIV viral load at MCNS diagnosis (copies/ml)	8029	0	0	0	0	0	1019	47,263
Previous history of opportunistic infection	No	No	No	Pneumocystosis	No	No	*M.avium* pulmonary infection	No
HBV infection	No	Yes	No	No	No	Yes	Yes	No
HCV infection	No	No	No	No	No	Yes	No	No
Proteinuria (g/24 h)	6.3	12	3.86	14.8	11	3.33	3.37	9
Serum albumine (g/L)	9.5	24	29.5	12	8	29	20	7.7
Serum creatinine (μmol/L)	109	130	77	62	110	61	134	79
AKI stage (KDIGO)	1	1	No	No	No	No	2	No
Hematuria (cells/ml)	0	34,000	0	34,000	13,000	0	500,000	0

*F* female, *M* male, *HT* hypertension, *NA* data not available, *MCNS* Minimal Change Nephrotic Syndrome, *AKI* acute kidney injury

The HAART regimen prescribed at the time of renal biopsy are listed in Table 2. MCNS diagnosis led to changes in HAART regimen in three patients (patients 2, 3 and 7). The main cause of modifications to the HAART regimen were the occurrence of AKI and prior administration of a HAART agent known to be potentially nephrotoxic (tenofovir in two patients).

**Table 2:** Change in highly active antiretroviral therapy (HAART) following MCNS diagnosis

Patient	HAART before MCNS diagnosis	Change in HAART at the time of MCNS occurrence
1	EVILTEGRAVIR COBICISTAT TENOFOVIR EMTRICITABINE	No change
2	EMTRICITABINE TENOFOVIR ETRAVIRINE	ABACAVIR LAMIVUDINE ETRAVIRINE
3	RALTEGRAVIR TENOFOVIR NEVIRAPINE	RALTEGRAVIR NEVIRAPINE
4	EMTRICITABINE TENOFOIR RILPIVIRINE	No change
5	LAMIVUDINE ZIDOVUDINE LOPINAVIR	No change
6	RALTEGRAVIR ETRAVIRINE	No change
7	ABACAVIR LOPINAVIR ETRAVIRINE	ABACAVIR LAMIVUDINE DIDANOSINE LOPINAVIR
8	None	None (HAART introduction 4 years after diagnosis)

### In situ hybridization for HIV-1 RNA detection and immunohistochemistry studies of CMIP expression on renal biopsy specimens

**Fig. 1:**
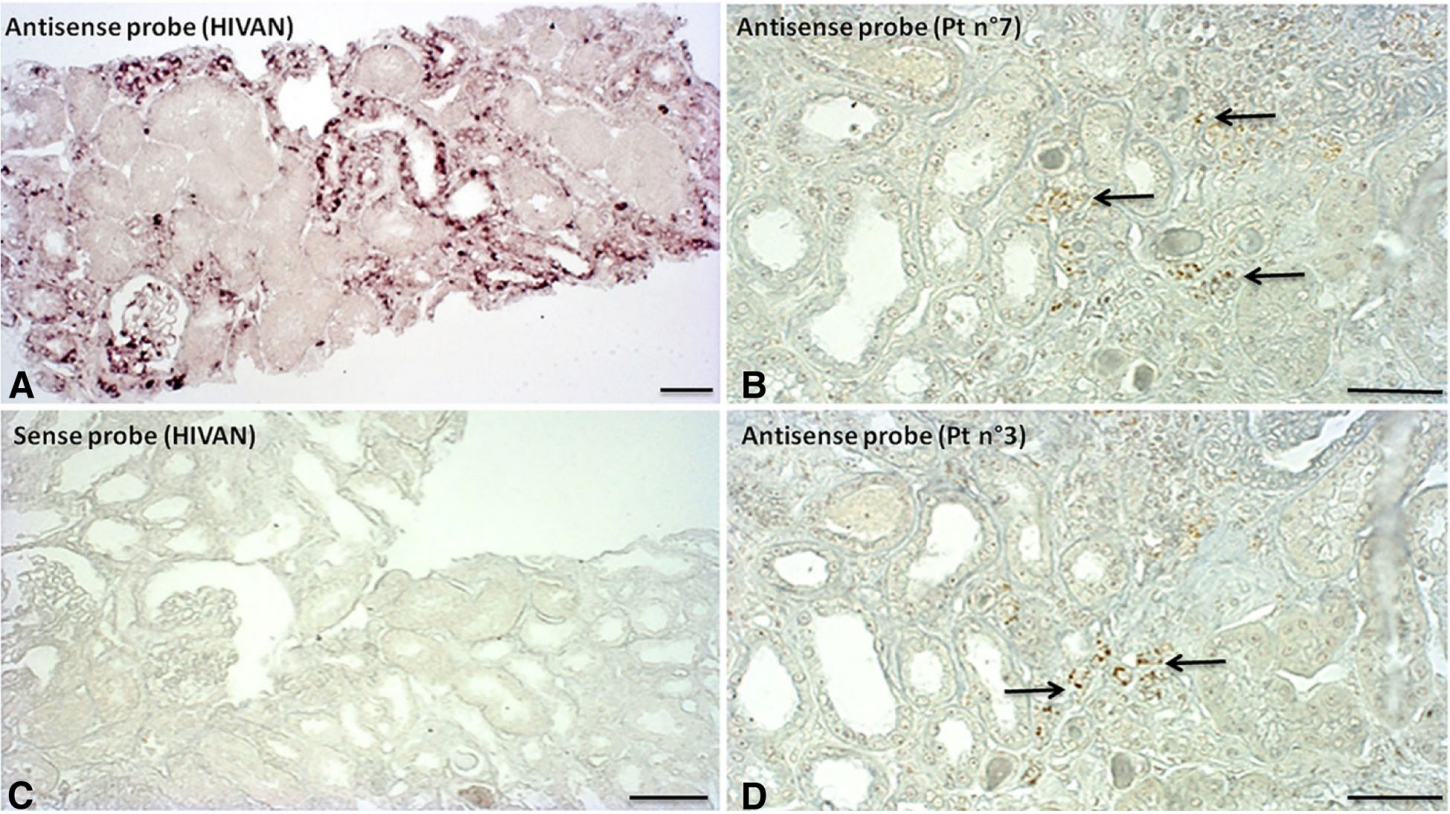
Detection of HIV mRNA in renal biopsy specimens by in situ hybridization (ISH). Representative ISH of HIV-1 RNA with antisense and sense probe (negative control) for patients with HIVAN (**a** and **c**) and for two patients with MCNS occurring in a context of HIV infection (**b** and **d**). In patients with HIVAN, antisense probe hybridization yields a positive signal for tubular epithelial cells and some glomerular cells (1**a**). A sense riboprobe was used as a negative hybridization control in serial sections (1**c**). No staining was detected in patients with MCNS in the absence of HIV infection (data not shown), whereas rare positive tubular cells (arrows) were observed in the absence of glomerular staining in two of four patients with MCNS in a context of HIV infection (**b** and **d**). Scale bar, 50 μm

Renal biopsy findings, including the results of light microscopy and immunofluorescence study were unremarkable. None of the eight patients had interstitial inflammatory infiltrate or acute tubular lesions. We investigated whether MCNS was associated with direct glomerular HIV infection, by analyzing HIV-1 RNA levels by in situ hybridization (ISH) on renal biopsies from four of the eight patients (patients 2, 3, 5, and 7). Unfortunately, renal biopsy specimens were not available for the 4 other patients*.* ISH with an antisense probe revealed the presence of HIV-1 RNA in the tubular and glomerular cells of control patients with HIVAN (*n* = 2), whereas no signal was detected with the sense probe (negative control) (Fig. 1 a and c). No HIV signal was obtained on renal biopsy specimens from control patients with idiopathic MCNS without HIV infection (n = 2) (data not shown)*.* In two patients with MCNS occurring in a context of HIV infection (patients 2 and 5), no positive cells were detected on renal biopsies. By contrast, in the two others patients (patients 3 and 7), ISH with an antisense probe revealed the presence of HIV-1 RNA in a very small number of tubular cells (Fig. 1b and d), despite that viral load was below the detection threshold for patient 3. No signal was detected in podocytes.

**Fig. 2:**
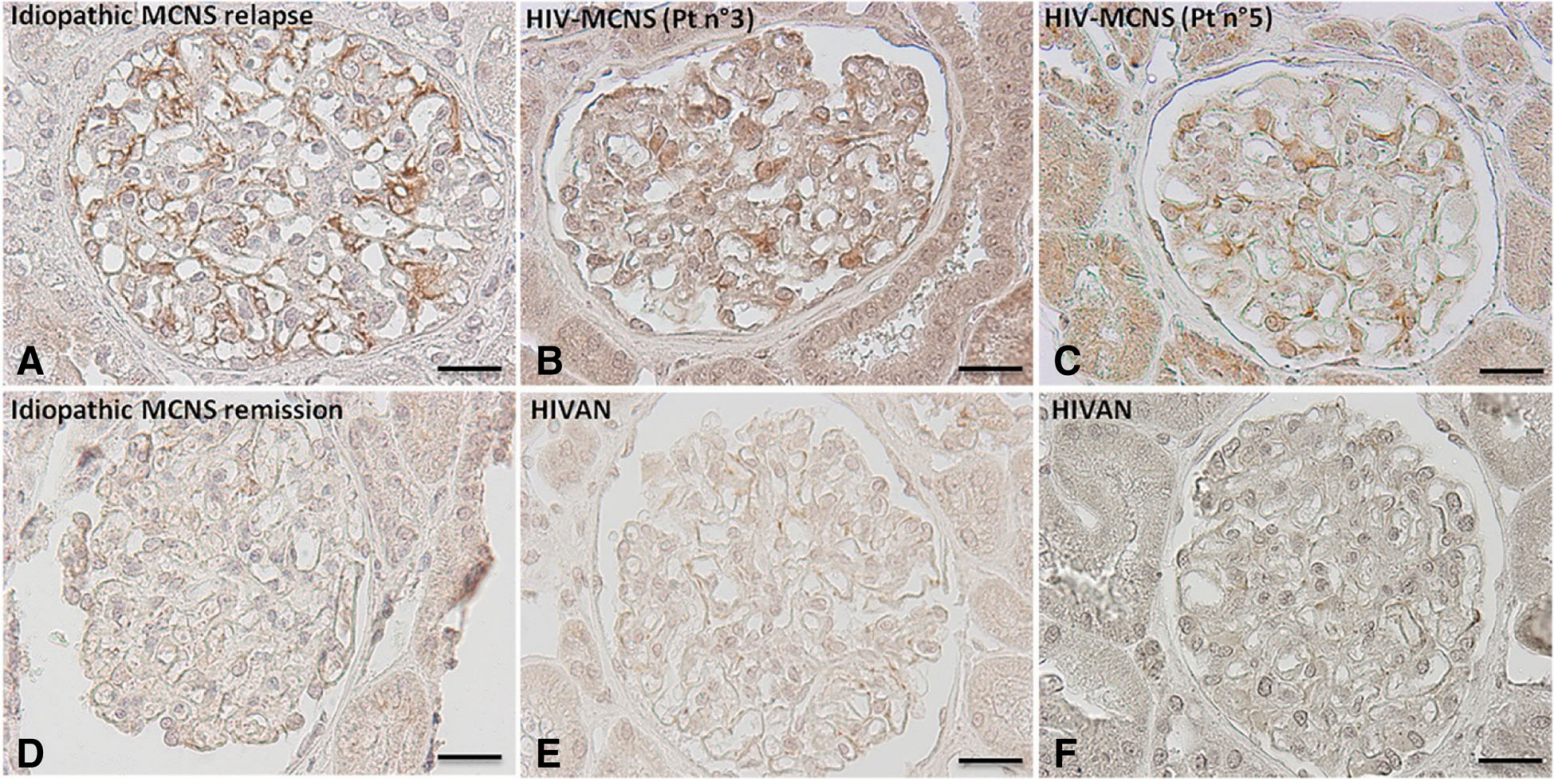
CMIP expression on renal biopsy specimens from patients with MCNS in a context of HIV infection. CMIP is induced in the podocytes of patients with idiopathic MCNS relapse (biopsy at the time of the first episode) (**a**), but it is expressed at only very low levels during remission (**d**). Two representative cases from patients with MCNS in a context of HIV infection are shown, displaying strong staining with anti-CMIP antibody (**b**, **c**). By contrast, immunohistochemical studies of CMIP levels revealed very weak signals on the glomeruli of patients with HIVAN (**e**, **f**). Scale bar, 50 μm

We then used immunohistochemistry methods to assess the expression of CMIP on renal biopsies from the same four patients with MCNS in a context of HIV infection. As observed in patient with idiopathic MCNS relapse on the renal biopsy performed during the initial episode (Fig. 2a), high levels of CMIP expression on podocytes were observed in patients with MCNS and HIV infection (Fig. 2b and c). By contrast, CMIP expression was very weak in the glomeruli of control patients with HIVAN, yielding a signal similar to that observed for a patient with MCNS in remission (Fig. 2 d, e and f).

### Treatment and outcome of MCNS occurring in a context of HIV infection

At the time of MCNS diagnosis, six patients received steroids as a first-line treatment (Table 3). Five of these patients displayed CR of nephrotic syndrome, whereas no data were available for one patient, who was lost to follow-up (patient 7). Two patients displayed spontaneous remission without specific treatment (CR in patient 3 and PR in patient 6). Three of the patients successfully treated with steroid displayed steroid-dependent MCNS (patients 2, 4 and 5), necessitating long-term steroid therapy. Another two patients (patients 1 and 3) suffered at least one relapse during follow-up, with the first relapse occurring nine and eleven months, respectively, after MCNS diagnosis. One patient (patient 1) was successfully retreated with steroids alone for a single relapse. Second-line treatment was initiated in four patients, because of steroid dependence (patients 2, 4 and 5) or frequent relapses (patient 3). Rituximab was used for second-line treatment in three patients (patients 2, 3 and 4). Patient 3 displayed a spontaneous remission during the initial episode. During the first relapse, nephrotic syndrome occurred simultaneously with a cerebral thrombophlebitis requiring immediate therapeutic intervention. Nevertheless, she declined to be treated by high dose of steroids (1 mg/kg) due to potential side effects and clinicians decided to treat this relapse with low dose of steroids (0.5 mg/kg) in association with Rituximab administration. Subsequently this patient had two other relapse requiring Rituximab therapy. Patient 5 was also given rituximab, due to multiple relapses despite triple therapy including steroids, cyclosporine and mycophenolate mofetil (MMF). Rituximab treatment led to sustained remission in two patients (patients 2 and 4) whereas the other two patients (patients 3 and 5) suffered relapses seven and four months, respectively, after rituximab treatment. At the end of follow-up (median follow-up of 20 months; range: 9 to 111 months), all patients for whom data were available (7/8) were considered to be in remission (2 PR and 5 CR; Table 3), with a mean eGFR of 91 mL/min/1.73 m^2^. At the time of the last follow-up evaluation, four patients (patients 1, 2, 3 and 5) were on specific treatment for MCNS (steroids). Infectious episodes occurred in two patients on rituximab: pneumonia in patient 2, and two episodes of pyelonephritis and one of *Clostridium difficile* colitis in patient 3.

**Table 3:** MCNS treatment and outcome

Patient	1	2	3	4	5	6	7	8
First-line treatment	Steroids	Steroids	Spontaneous remission	Steroids	Steroids	Spontaneous remission	Steroids	Steroids
CR	Y	Y	Y	Y	Y	N	NA	Y
PR	–	–	–	–	–	Y	NA	–
Steroid-dependence (dose of steroid at the time of relapse)	N	Y (10 mg/d)	N	Y (5 mg/d)	Y (5 mg/d)	N	NA	N
Steroid resistance	N	N	N	N	N	N	NA	N
Number of relapses during follow-up	1	2	3	2	> 6	0	NA	0
Time between MCNS diagnosis and first relapse (months)	9	3	11	1	8	–	–	–
Second line treatment	Steroids	Steroids + Rituximab	Steroids + Rituximab	Steroids + Rituximab	Steroids + CsA + MMF + Rituximab	–	–	–
Follow-up (months)	13	20	68	14	111	9	NC	91
Status at last follow-up visit	CR	PR	CR	CR	CR	PR	NA	CR
Proteinuria (g/24 h) at last follow-up visit	0.1	1.25	0.12	0.1	0.28	0.89	NA	0.1
Serum albumine (g/L) at last follow-up visit	36.2	37.2	40	37	31	43	NA	40
Serum creatinine (μmol/l) at last-follow-up visit	78	95	138	64	66	71	NA	109
eGFR CKD-EPI (ml/min/1.73m^2^) at last follow-up visit	99	73	39	104	120	99	NA	108

*Y* Yes, *N* No, *CsA* cyclosporine A, *CR* complete remission, *PR* partial remission, *NA* data not available

## Discussion

HIV-related renal diseases include a large diversity of pathological entities, but the most relevant glomerular disorders observed in these patients are HIVAN and immune complex-mediated glomerular disease [11, 18]. The occurrence of MCNS in the setting of HIV infection has never been studied in detail and the main characteristics of this association remain to be determined. Over a 16-year period, we could find only eight patients with MCNS in a context of HIV infection, suggesting that this association may be fortuitous rather than indicative of a direct relationship. However, our study was not designed to accurately assess the prevalence of MCNS in HIV infected patients.

Genetic factors, including *APOL1* variants, have recently emerged as key factors potentially increasing susceptibility to HIVAN in HIV-infected patients of African ancestry [19, 20]. In our study, *APOL1* variants were not systematically checked, but the distribution of Caucasian and African patients in our cohort suggests that this was not a susceptibility factor for this association. Moreover a recent study suggested that two risk allele genotype is uncommon in patients with MCNS and mainly found in patient with FSGS steroid resistant nephrotic syndrome [21]. In a retrospective study, Lescure et al. described changes in the pattern of glomerular lesions in HIV patients [14]. They demonstrated that the incidence of HIVAN had strongly decreased over time, since the introduction of HAART, and that classical FSGS was the leading causes of glomerular disease in HIV-infected patients. Lescure et al. found that MCNS was the main pathological lesion in four patients (4.5% of cases). Strikingly, seven patients from our cohort were on HAART and HIV infection was considered to be under control at the onset of nephrotic syndrome. These data indicate that MCNS was probably not due to severe immunodeficiency in these patients. MCNS occurred exclusively in one patient newly diagnosed with HIV infection, with no obvious difference in clinical, biological and histological presentation compared to other patients in whom HIV infection was well controlled. In this specific case, we can not rule out that HIV infection in the context of immune susceptibility or particular genetic background might contribute to trigger MCNS relapse, as was recently reported for EBV infection [22]. In three patients, the diagnosis of MCNS led to change the antiretroviral drugs used, due to potential tenofovir-mediated renal toxicity in two cases and AKI in one case. Tenofovir administration has been identified as a potential cause of tubular injury, sometimes associated with significant but non-nephrotic proteinuria [23]. However, none of our patients on this drug had typical proximal tubular dysfunction or pathological lesions suggestive of toxic tubular necrosis. In a study of 95 adult patients with idiopathic MCNS, Waldman et al. found that AKI was present at initial presentation or during a relapse episode in 25.2% of cases, mostly due to acute tubular injury or interstitial inflammation [24]. Three patients from our cohort displayed AKI according to KDIGO criteria but, in these cases, analyses of renal biopsies provided no evidence of interstitial inflammatory lesions or tubular injury, suggesting a prerenal origin of AKI.

We investigated the potential role of HIV infection in MCNS occurrence in this context, by performing ISH to determine whether the renal cells of these patients were infected*.* By contrast to HIVAN patients, in which large numbers of tubular and glomerular cells gave a positive signal, no HIV RNA was detected in the cells of two patients from our cohort with viral loads below the threshold of detection. Nevertheless, ISH detected a few tubular cells displaying positive staining, with a complete absence of staining in the glomerular area, in two other patients with MCNS in a context of HIV infection. In one case, HIV RNA was detected in the renal parenchyma, despite undetectable viremia. Winston et al. were the first to demonstrate, in a patient diagnosed with HIVAN that HIV-1 mRNA may persist in the renal epithelium even if viral RNA is undetectable in the plasma, following the initiation of HAART [25]*.* Consistent with this hypothesis, Canaud et al. found that kidney graft parenchyma may serve as a viral reservoir after kidney transplantation [17]. Another key finding of our study is the induction of CMIP expression in the podocytes of patients with MCNS in a context of HIV infection. CMIP upregulation has been described in renal biopsies from patients with idiopathic MCNS, FSGS and membranous nephropathy, whereas CMIP is not generally expressed in inflammatory and proliferative glomerular diseases, such as IgA nephropathy or active lupus nephritis [6, 26, 27]. In vivo and in vitro experiments have shown that CMIP plays a crucial role in podocyte dysfunction, by inactivating the nephrin and Akt signaling pathways responsible for altering podocyte signaling and cytoskeleton remodeling, an important event in the induction of proteinuria [6]. In addition, experimental data from several models of podocyte injury suggest that targeting CMIP with specific inhibitors taken up by podocytes may be a promising future therapeutic approach for some glomerular diseases associated with the overproduction of CMIP in podocytes [6, 26]. By contrast, in patients with HIV infection, pathophysiological processes involved in HIVAN seems to be closely related to viral integration into target cells. Thus, the expression of HIV proteins, such as Nef, in particular, in podocytes, leads to the activation of STAT3 signaling, which seems to be a key factor in HIVAN pathogenesis [28, 29]. Thus, the absence of HIV RNA in glomerular cells and the induction of CMIP in podocytes from patients with MCNS in a context of HIV infection suggest that the occurrence of MCNS in individuals with HIV infection may be coincidental and unrelated. As MCNS may primarily result from an immune system disorder [2], chronic HIV infection may triggers immune disturbances in some patients, promoting podocyte dysfunction.

In accordance with current recommendations for the treatment of initial episodes of idiopathic MCNS in adult patients [1], first-line treatment with steroids was initiated in six patients, leading to CR in five cases (the remaining patient being lost to follow-up). Two of the patients in our cohort presented spontaneous remission, consistent with the findings of Maas et al., who reported the occurrence of spontaneous progressive remission in 10% of patients not receiving specific therapy [30]. After a median of 20 months of follow-up, five of the seven patients for whom data were available presented relapses of MCNS (including three patients with steroid-dependent MCNS). Strikingly, rituximab was the treatment of choice most frequently prescribed for patients with steroid-dependent MCNS. It was also prescribed for one patient with frequent relapses. Rituximab has recently emerged as a promising treatment for steroid-dependent nephrotic syndrome [31–33]. In a context of HIV infection, Rituximab is primary used for the treatment of patients with HIV related lymphoma and Multicentric Castleman’s disease [34]. However, Rituximab may increase the risk of infectious episodes in HIV patients. Our retrospective study was not designed to investigate the safety and tolerance of Rituximab in this population. In our cohort, non-fatal bacterial infections occurred in two patients on rituximab. Given that rituximab administration has been associated with an increase in the risk of hepatitis B reactivation, opportunistic infections and polyomavirus JC-related progressive multifocal leukoencephalopathy [35], this treatment should probably be used with caution in HIV-infected patients.

## Conclusions

MCNS may be observed in HIV patients, but seems to be a rare glomerular disease in these patients. Our pathology studies suggested that HIV infection was not directly involved in podocyte dysfunction and that CMIP induction, through unknown mechanisms, might play a key role in podocyte dysfunction and the occurrence of proteinuria. First-line treatment for MCNS in patients with HIV infection should include steroids, but, given the high incidence of MCNS relapse and steroid dependence, the potential contribution of rituximab to treatment should be investigated further.

## Abbreviations

AKI: Acute kidney injury
*APOL1*: Apolipoprotein L1
CKD-EPI: Chronic kidney disease epidemiology collaboration
CMIP: C-maf induction protein
CR: Complete remission
eGFR: estimated glomerular filtration rate
FSGS: Focal segmental glomerulosclerosis
HAART: Highly active anti retroviral therapy
HIV: Human immunodeficiency virus
HIVAN: Human immunodeficiency virus associated nephropathy
ISH: In situ hybridization
KDIGO: Kidney disease improving global outcome
MCNS: Minimal change nephrotic syndrome
MMF: Mycophenolate mofetil
NS: Nephrotic syndrome
PR: Partial remission
RNA: Ribonucleic acid
